# Identification of novel immune cell signature in gastroesophageal reflux disease: altered mucosal mast cells and dendritic cell profile

**DOI:** 10.3389/fimmu.2023.1282577

**Published:** 2023-11-30

**Authors:** Ahsen Ustaoglu, Fatema Arif Daudali, Manfredi D’afflitto, Stephen Murtough, Chung Lee, Estefania Moreno, Diana C. Blaydon, David P. Kelsell, Daniel Sifrim, Philip Woodland, Madusha Peiris

**Affiliations:** ^1^ Blizard Institute, Barts and The London School of Medicine and Dentistry, Queen Mary University of London, London, United Kingdom; ^2^ Royal London Hospital, Barts National Health Service (NHS) Health, London, United Kingdom

**Keywords:** GERD, heartburn, mast cells, dendritic cells, neuro-immune, RNA-seq

## Abstract

**Introduction:**

Heartburn pathogenesis in GERD remains incompletely understood. We aimed to identify differences in the immune cell signature and sensory mucosal markers between reflux phenotypes and healthy asymptomatic subjects.

**Methods:**

Thirty-seven patients with heartburn symptoms were phenotyped endoscopically and with objective reflux studies into erosive reflux disease (ERD) (N=10), nonerosive reflux disease (NERD) (N=9), functional heartburn (FH) (N=9), and Barrett’s esophagus (BO) (N=9). Bulk mRNA-sequencing(RNA-seq) was conducted on RNA extracted from endoscopic biopsies, and immune cell deconvolution analysis was performed using CIBERSORT. RNA-seq findings were validated by immunofluorescent staining for CD1a, nerve growth factor (NGF), and mast cell tryptase in corresponding patient biopsies.

**Results:**

Transcriptomic analysis detected higher mast cell abundance in BO, ERD, and NERD compared to healthy controls (*p*<0.05), with decreased dendritic cell infiltration in BO, ERD, and NERD patients compared to healthy controls and FH patients. CD1a-positive dendritic cell infiltration was significantly higher in the healthy esophageal mucosa at protein level compared to BO (*p*=0.0005), ERD (*p*=0.0004), and FH patients (*p*=0.0096). Moreover, NGF co-expression on mast cells in GERD patients was significantly higher than in healthy controls (*p*=0.0094).

**Discussion:**

The mucosa in patients with GERD had a significant increase in NGF expression on mast cells, suggesting an upregulation of signalling for neuronal sprouting in GERD. Moreover, decreased dendritic cell abundance in GERD esophageal mucosa may play a role in reduced oral tolerance and development of subsequent immune responses which may participate in esophageal sensitivity.

## 1 Introduction

Gastroesophageal reflux disease (GERD) is among the top 3 outpatient diagnostics in the United States, and refractory symptoms including heartburn and regurgitation persist in over 30% of patients who do not respond to acid suppressant therapy (1–4). Moreover, heterogeneity exists in symptom perception between GERD patients. While the mechanisms underlying heartburn in erosive reflux disease (ERD) can be attributed to macroscopic mucosal inflammation visible at endoscopy, molecular mechanisms underlying heartburn pathogenesis are less clear, particularly in the absence of macroscopic mucosal injury in over 60% of GERD patients who are diagnosed with non-erosive reflux disease (NERD) (1, 5). PPI-refractory NERD patients demonstrate hypersensitivity to acid and, occasionally, saline perfusion, compared with their erosive counterparts and healthy controls (6–8). In contrast, many Barrett’s esophagus (BE) patients do not present heartburn symptoms despite having had years of pathological acid reflux, while functional heartburn patients (FH) perceive heartburn despite having no association with reflux events (9).

Identifying the molecular signature of the esophageal mucosa specific to the discrepancy of symptoms observed between GERD patients with similar levels of acid exposure will assist in the development of novel treatments. The histologic finding of dilated intercellular spaces, a morphologic measurement of a “leaky” epithelium that allows easy passage of noxious refluxate, may indicate impaired mucosal integrity in NERD patients and contribute to heartburn perception (10). We recently identified the expression of transient receptor potential vanilloid subfamily member-1 (TRPV1) on superficial sensory nerves in patients with NERD, alongside increased epithelial cell expression of acid-sensing ion channel-3 in ERD and NERD, collectively highlighting the potential mucosal mechanisms for heartburn sensation in different reflux phenotypes (11).

The immune cell signature of the esophageal mucosa in heartburn patients without overt inflammation has not previously been characterized. Based on what is known about symptom-based conditions in other gastrointestinal tissues, neuroimmune interactions may also play an important mechanistic role in symptom generation, given that sensory neurons have also been shown to signal to tissue-resident innate immune cells including dendritic cells during the early phases of inflammation (12, 13). We sought to identify the immune cell signature of GERD phenotypes underlying the differences in the mucosal pathogenesis of heartburn sensation and, thus, the potential future therapeutic implications, by first using a bulk RNA-sequencing approach followed by localization studies of the esophageal mucosa from GERD patients and asymptomatic subjects.

## 2 Materials and methods

### 2.1 Study subjects

All patients were prospectively recruited (2019–2022) following informed consent and were required to have a clinical history of problematic heartburn requiring investigation with a clinical referral for endoscopic examination. Adults of either gender (18–70 years old) with symptoms of at least moderate heartburn more than 3 times per week were included. Patients were excluded if they 1) were pregnant, 2) had severe upper gastrointestinal motility disorders, 3) were hypersensitive to local anesthetic, 4) took coagulopathy or concurrent anticoagulant medication, and 5) had any other medical condition that would make it unsafe for the patient to participate. All patients filled out the validated Reflux Disease Questionnaire during consent (**Supplementary Table 1**).

Four patient groups were studied: NERD, ERD, BE, and FH. Patients were recruited from the Royal London Hospital (Queen Mary University of London). BE patients were recruited from a dedicated BE endoscopic surveillance list at the Royal London Hospital. The study was granted ethical approval by the NRES Committee London-Queensquare (Study reference: 19/LO/1506).

Patients underwent endoscopy +/− wireless ambulatory reflux monitoring. All patients (except those with known Barrett’s esophagus) had stopped PPI treatment for >7 days before endoscopy and reflux testing. Post-procedure, patients were divided into clinical phenotypes according to the definitions detailed below.

#### 2.1.1 Erosive reflux disease patients

Symptomatic patients with at least LA grade B esophagitis at endoscopy were included in the ERD group. Five distal esophageal mucosal biopsies were taken per patient from non-eroded mucosa 3 cm above the gastroesophageal junction.

#### 2.1.2 Non-erosive reflux disease and functional heartburn patients

In symptomatic patients who showed no visible esophageal injury on endoscopy, five distal esophageal mucosal biopsies were obtained per patient (3 cm above the gastroesophageal junction). In the same session, an endoscopically sited wireless intraesophageal pH sensor capsule was placed [systems used: OMOM, Jinshan Science & Technology (Group) Co. Ltd., Chongqing, China; or Bravo, Medtronic, Shoreview, MN, USA). A 96-h pH recording was performed.

Patients with pathological acid exposure (>4.2% over the study period) on analysis of their reflux studies qualified for a diagnosis of NERD. Patients whose reflux testing studies did not meet pathological acid exposure criteria and had negative reflux/symptom association were diagnosed with FH and included in the study. Patients with physiological acid exposure and positive reflux/symptom association (i.e., hypersensitivity patients) were not included due to insufficient patient numbers.

#### 2.1.3 Barrett’s esophagus patients

Adults undergoing routine endoscopic surveillance for known BE with heartburn symptoms or screening for suspected BE following previous endoscopic diagnosis of ERD were prospectively recruited. In keeping with treatment guidelines for BE patients, PPI treatment was not stopped prior to endoscopy. Patients with clear visualization of columnar epithelium ≥1 cm above the gastroesophageal junction on endoscopy and histopathologic recognition of intestinal metaplasia were diagnosed with BE. In addition to clinical surveillance biopsies, five distal esophageal mucosal biopsies were taken from the squamous mucosa 1 cm above the squamocolumnar junction, away from the BE segment, for the purposes of this study.

#### 2.1.4 Healthy control group

Data from patients with ERD, NERD, FH, and BE were compared with the data from a group of healthy and asymptomatic volunteers. Fourteen asymptomatic volunteers (aged 18–80) were recruited and studied (**Supplementary Table 1**). None had a history of gastrointestinal symptoms, and none had a history of anti-reflux medications. All healthy controls (HCs) had a Reflux Disease Questionnaire score of 0. Healthy controls were excluded if they 1) had previous upper GI surgery, 2) had severe upper GI motility disorders, 3) were pregnant, 4) were taking coagulopathy or concurrent anticoagulant medication, and 5) had any severe midface trauma or recent nasal surgery.

All subjects had a normal esophageal appearance on endoscopy. Five distal esophageal biopsies were obtained per volunteer (3 cm above the gastroesophageal junction) at the Royal London Hospital. Distal esophageal biopsies of these HCs were prepared and analyzed in an identical fashion to the patient biopsies used in this study.

In total, biopsies from 75 patients reporting with heartburn were analyzed. For RNA sequencing studies, we analyzed 37 patient biopsies. Immunohistochemical analyses were conducted on a minimum of 10 representative patient biopsies per phenotype for each panel of staining (**Table 1**).

**Table 1:** Demographic data of study subjects.

Phenotype	Number of participants studied for RNA sequencing analysis	Mean age (years)	Age range (years)	Female:male
NERD	9	44	28–53	3:6
ERD	10	46	21–74	2:8
FH	9	41	20–71	3:6
BE	9	54	31–75	4:5
HC	8	27	20–35	6:2
Phenotype	Number of patients studied for IF analysis	Mean age (years)	Age range (years)	Female:male
NERD	11	52	30–71	5:6
ERD	23	45	22–61	7:16
FH	18	46	22–70	10:8
BE	19	60	32–78	8:11
HC	10	31	20–70	6:4

### 2.2 Biopsy processing

Of the five biopsies, three were orientated and fixed in 4% paraformaldehyde (PFA) for 3 h, followed by cryoprotection in 30% sucrose in phosphate-buffered saline (PBS) for 24 h at +4°C. Fixed tissue was embedded in optimum cutting temperature compound (Sakura Tissue-Tek, Torrance, USA, Cat. No. 4853), and 4 serial (10 µm) sections were cut perpendicular to the mucosal surface on a cryostat (Leica 180UV) and mounted on positive-charged glass slides (Thermo Scientific, Waltham, Massachusetts, USA, J1800AMNZ).

Two of the five biopsies taken per patient at endoscopy were placed in RNAlater and stored at −80°C until RNA extraction.

### 2.3 Bulk mRNA sequencing

RNA was extracted from a representative number of GERD and HC esophageal biopsies (**Table 1**) stored in RNAlater at −80°C until use. RNA was extracted using the RNeasy mini kit as per the manufacturer’s instructions, including on-column DNAse I digestion (both from Qiagen, Hilden, Germany). Subsequent processing of RNA samples took place at the Blizard Genome Centre where eluted RNA was quantified using a NanoDrop™ spectrophotometer (Thermo Fisher 2000/2000c). The quality and integrity of the total RNA were assessed using Bioanalyser 2100 (Agilent, Santa Clara, USA) and assigned an RNA integrity (RIN) score. Only samples with RIN >8 were used to prepare libraries for RNA sequencing.

Total RNA (100 ng) was used to prepare directional mRNA libraries using the NEBNext Ultra II Directional RNA library preparation kit multiplex oligos for Illumina (New England BioLabs, Ipswich, Massachusetts, USA, Cat. No. 10032630). Libraries were then quantified prior to pooling using a Qubit 2.0 fluorometer and qualified (Agilent TapeStation system) using D1000 ScreenTape and reagents (Agilent). Libraries were diluted and pooled, and pooled libraries were requantified prior to sequencing (Qubit 2.0 and Agilent TapeStation systems) using HS D1000 reagents (Agilent). Finally, mRNA libraries were sequenced on the NextSeq500 system (Illumina, San Diego, California, USA) by the Blizard Genome Centre.

### 2.4 RNA sequencing data analysis

Sequencing analysis was conducted using Partek Flow^®^ software. FASTQ files were demultiplexed and underwent pre-alignment QC to ensure that the collected data did not have any obvious systematic errors before alignment. Next, Spliced Transcripts Alignment to a Reference (STAR) was used to align sequenced reads to the hg38 human genome (14). Adapter sequence overrepresentation was insignificant, so trimming was not necessary. Post-alignment QC was performed to check the quality of alignment. All samples had more than 97% alignment to the genome. The total number of reads was more variable, but most samples had more than 17 M reads (**Supplementary Table 1**). There was no parameter for removing outliers based on QC metrics.

Principal component analysis (PCA) was then performed including the first two principal components following batch effect correction. The PCA shows the similarity between healthy controls (**Supplementary Figure 1A**), where they are spatially arranged close to one another. Samples NE8 (FH), SH061119 (BE), and RC110320 (BE) appeared to be outliers as they were spatially dissimilar to the other GERD samples. These samples were further evaluated using a selection of Barrett’s segment (*SOX9*, *MUC5AC*) and stromal collagen genes (*COL3-6A*) to check the significant expression of these genes among any of the samples (**Supplementary Figure 1D**). The three outliers identified by PCA had significantly higher expression of stromal collagen genes (NE8) and Barrett’s segment genes (SH061119 and RC110320), and were excluded from downstream analysis (**Supplementary Figure 1D**). **Supplementary Figure 1C** shows PCA once outliers have been removed.

Aligned genes were normalized using median ratio for DESeq2 on Partek Flow. Differentially expressed genes between phenotypes were calculated using the Wald test in DESeq2 R package (15) with an FDR-adjusted *p*-value of less than 0.01. The most biologically significantly differentially expressed genes were visualized as hierarchical clustering heatmaps on Partek Flow^®^.

Gene set enrichment analysis of FDR-filtered differentially expressed genes was performed on Partek Flow^®^ with a 0.01 *p*-value cutoff. R was used to visualize the most significantly biologically enriched gene functions. Metascape and Cytoscape were used to visualize functionally enriched gene ontologies (GO) and compare GSEA results (16).

### 2.5 Cellular deconvolution

Deconvolution analysis for quantification of relative levels of distinct cell types per sample was carried out on normalized counts using CIBERSORT (17). Bulk gene signatures of GERD patient phenotypes and HCs were grouped into six different immune cell categories.

### 2.6 Quantitative real-time polymerase chain reaction

RNA was extracted from esophageal mucosal biopsies stored in RNAlater™ (Sigma, St Louis, Missouri, USA, Cat. No. R0901-100ml) solution using RNeasy Mini Kit (Qiagen, Cat. No. 74016) and DNAse-treated (Qiagen, Cat. No. 79254) according to the manufacturer’s instructions. Eluted RNA was quantified using the NanoDrop™ spectrophotometer (Thermo Fisher 2000/2000c), and only samples with >100 ng/µl of RNA were used for the qPCR experiments. DNA was synthesized using the QuantiTect reverse transcription kit (Qiagen, Cat. No. 205310). Quantitative real-time polymerase chain reaction (qRT-PCR) was performed on the AB7300 Real-Time PCR system (Applied Biosystems) using the QuantiFast SYBR green PCR kit (Qiagen, Cat. No. 204056). QuantiTect Primer Assays (Qiagen) were used for the *18S* (QT00199367), *NGF* (QT00001589), and *CXCL8* (QT00000322) genes. Relative gene expression was calculated using the 2^−ΔΔCT^ method as described previously (18).

### 2.7 Immunofluorescence staining

Sections were air-dried for 1 h, washed with PBS to remove the embedding medium, and serum-blocked to remove non-specific binding (Abcam, Cat. No. ab64226). Sections were then incubated with a combination of primary antibodies for the detection of immune cells, nociceptive sensory nerves, or inflammatory cytokine receptors at +4°C for 16–18 h. The following conditions were used for each primary antibody: protein gene product 9.5 (PGP9.5, a pan-neuronal marker used to identify afferent nerves) (1:200 dilution, polyclonal rabbit anti-human, Dako, Cat. No. Z5116), nerve growth factor (NGF, 1:200 dilution, monoclonal rabbit anti-human, Abcam, ab52918), mast cell tryptase (1:400 dilution, monoclonal mouse anti-human, Dako, Cat. No. M7052), and CD1a (used as an activated dendritic cell marker) (1:200 dilution, monoclonal mouse anti-human, Dako, Glostrup, Denmark, Cat. No. M3571). Following overnight incubation, sections were washed 3 times (10 min/wash) in PBS, and a secondary antibody was applied (donkey anti-mouse 488 nm and donkey anti-rabbit 568 nm, Invitrogen, Thermo Fisher Scientific, ab175470 and ab150105, 1:400 concentration) for 1 h. Sections were then washed 3 times in PBS and mounted with Vectashield HardSet antifade mounting medium with DAPI fluorescent stain (4′,6-diamiidino-2-phenylindole; Vector Laboratories, Newark, California, USA, H-1500) and a 0.16–0.19-mm coverslip (Thermo Fisher Scientific, 22X30-1.5).

Negative control slides were prepared with the primary antibody omitted but the secondary antibody was applied and did not show positive labeling (data not shown). Specific binding of immune cell markers and NGF antibodies was confirmed using inflammatory bowel disease (IBD) colon tissue. These sections showed specific binding of CD1a and tryptase and NGF, where cellular expression was observed between crypts in the colon and positive cells often co-localized with pan-leukocyte marker CD45 (data not shown). PGP9.5-immunoreactive structures were identified, and nerve distribution was characterized as previously described (11). All microscopy was performed using a Leica DM4000 Epi-fluorescence microscope, except when specified as being acquired using a Zeiss 880 laser scanning confocal microscope.

### 2.8 Image analysis

The distance of afferent mucosal nerve endings was confirmed in terms of the number of cell layers from the fiber to the luminal surface as previously described (11). NGF/mast cell tryptase co-expression was quantitatively assessed on Fiji using the JaCOP plugin, Manders’ coefficient (a Mander’s coefficient of 1.0 indicates 100% overlap, while 0 indicates no overlap between the channels assessed). For immune cell counting, the percentage of cells positive for the marker of interest was calculated relative to the total number of DAPI-positive cells. Cells were counted automatically using the “analyse particles” tool of Fiji. Five images were quantified per sample, and a meal cell count per sample was calculated. Submucosal cells were excluded from the analysis.

### 2.9 *Ex-vivo* biopsy assay

Three esophageal mucosal biopsies from a total of three ERD patients and five healthy volunteers were taken at endoscopy and immediately transported on ice in Dulbecco’s modified Eagle’s medium (DMEM) liquid (high glucose) with GlutaMAX I (Life Technologies, Cat. No. 31966021) supplemented with 0.4% penicillin/streptomycin (50 U/ml) (Sigma, Cat. No. P4333-20ML) and processed within 15 min. Biopsies were individually weighed. In a sterilized hood, biopsies were carefully placed in warm DMEM media in 96-well plates, ensuring minimal disruption to biopsies. Biopsies were incubated at 37°C with 95% O_2_ and 5% CO_2_ to allow normalization for 30 min. The plate was then taken out, the supernatant was carefully removed, and the aliquots were stored at −80°C until used for cytokine quantification as the “baseline” concentration. Wells with biopsies were replaced with fresh DMEM and placed on ice. Mucosal biopsies were sequentially and carefully orientated using a stereomicroscope to have an apical to basolateral polarity, thereby anchoring the submucosal aspect of the biopsy onto a 0.4-μm membrane in a Transwell insert (from 6-well Transwell plate). The luminal aspect of each biopsy was sequentially challenged with pH 7 (control), pH 5, and pH 2 for 5 min, washed with PBS, and placed back in their respective wells with DMEM media in a 96-well plate, and subsequently maintained at 37°C with 95% O_2_ and 5% CO_2_ overnight (18 h). The supernatant was then removed and aliquots were stored at −80°C.

### 2.10 Cytokine detection

Esophageal mucosal biopsies taken at endoscopy were challenged with pH 5 and pH 2 acid as described in the **Supplementary Methods**. Quantification of β-NGF was performed using a Bio-Plex Pro Human Cytokine Assay (171304090M, Bio-Rad). The assay plate was read using the xPONENT software on a MAGPIX detection system. The results file was extracted and analyzed in Bio-Plex Manager. The concentration in range for the baseline supernatant was subtracted from the sample post-pH challenge to give the concentration of cytokine release (pg/ml). Cytokine release between ERD and healthy controls and cytokine release with different pH conditions were compared by two-way ANOVA using GraphPad Prism 9.0. Values are presented as mean ± SD.

### 2.11 Statistical analysis

A two-way ANOVA test was used to compare the expression levels of the genes of interest between GERD patients and healthy controls and among GERD phenotypes. A one-way ANOVA test was used to analyze differences in quantitative levels of protein expression between GERD phenotypes. When ANOVA was positive, the Bonferroni test was used to identify which of the pairs was significantly different. Values are expressed as mean ± standard deviation (SD). GraphPad Prism 9.0 was used for the statistical analysis.

## 3 Results

### 3.1 Global transcriptome of GERD esophageal mucosa highlights differential immune regulation

**Figure 1:**
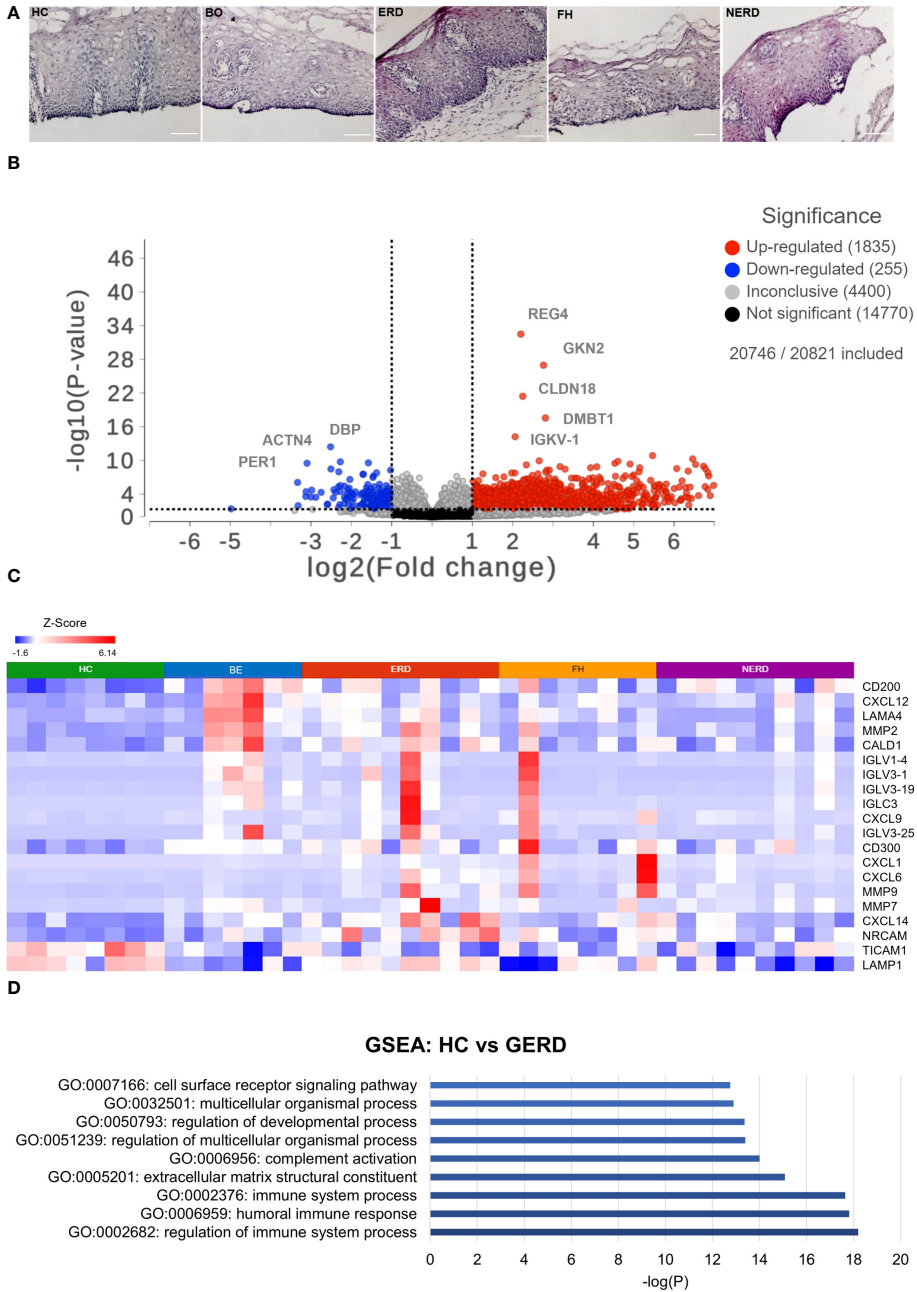
Global transcriptome highlights differential immune regulation of esophageal mucosa in gastroesophageal reflux disease (GERD). **(A)** H&E staining of fixed corresponding biopsies of samples included in the RNA sequencing (and immunofluorescence) studies highlights that all samples had an intact basal layer and papillary structures indicating an acceptable tissue thickness. Submucosa was occasionally seen in some samples, such as the representative ERD sample here. The scale bar represents 100 μm. **(B)** Volcano plot showing upregulated genes in GERD as log2-scaled fold change in red dots on the right of the graph and genes downregulated in GERD compared with healthy controls (HCs) on the left. Five hundred eighty-nine genes were upregulated in GERD, and 94 genes were downregulated in GERD compared with HCs. **(C)** Heatmap displaying the immune-related significantly DE genes between healthy controls and GERD with FDR *p* = 0.01. **(D)** Bar graph displaying the most biologically enriched gene ontology (GO) pathways from GSEA as log-scaled *p*-values (*p* < 0.01). HC: *N* = 8, Barrett’s esophagus (BE): *N* = 7, erosive reflux disease (ERD): *N* = 10, functional heartburn (FH): *N* = 8, non-erosive reflux disease (NERD): *N* = 9.

The corresponding fixed frozen biopsies from patients and healthy controls included in the RNA sequencing study were stained with hematoxylin and eosin (H&E) to determine sample viability. All samples had an intact basal layer of the epithelium and contained papillary structures indicating adequate tissue thickness across all patient phenotypes studied (**Figure 1A**).

To assess quantitative immune-relevant gene expression changes between the esophageal mucosa of healthy controls and GERD patients, differential expression (DESeq2) analysis was performed between healthy controls (*N* = 8) and GERD patients (*N* = 37). An FDR filter of <0.01 was applied and detected 979 differentially expressed (DE) genes between the normal esophageal mucosa and that of GERD patients, as shown in **Figure 1B**. GERD patients had 589 upregulated genes, while only 94 genes were downregulated in GERD compared with HCs (**Figure 1B**). The most significantly differentially expressed genes between HCs and GERD esophageal mucosa included the upregulation of immune-related genes such as *IGLC3*, *CXCL6*, and *MMP2* (**Figure 1C**). DESeq2 analysis also picked up genes related to the structural organization of the esophageal mucosa including *ACTN4* which participates in cytoskeletal reorganization and may induce hyperplasia often seen in ERD and NERD patients (**Supplementary Figure 2A**) (19, 20).

Gene set enrichment analysis (GSEA) was performed to infer important biological processes and molecular functions associated with DE genes between healthy and GERD esophageal mucosa. A total of 979 significantly DE genes were taken as input and were highlighted in 896 molecular pathways including regulation of the immune system and humoral immune response as statistically significative (*p* ≤ 0.01) (**Figure 1D**, **Supplementary Figure 2B**).

**Figure 2:**
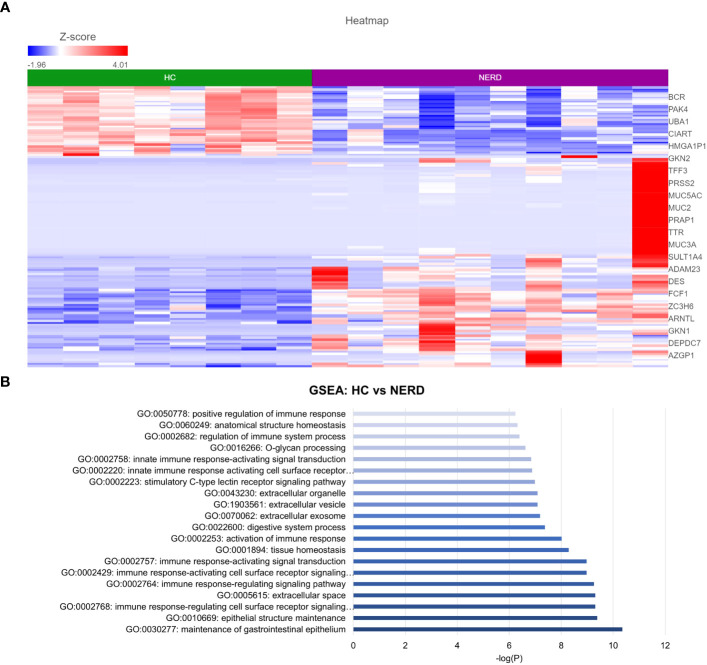
Differential gene expression in normal and NERD esophageal mucosa. **(A)** Heatmap displaying the top significantly differentially expressed (DE) genes between HC and NERD from a total of 137 significantly DE genes. **(B)** Bar graph of statistically significantly enriched biological pathways against log-transformed *p*-values (*p* < 0.01). HC: *N* = 8, NERD: *N* = 9.

**Figure 3:**
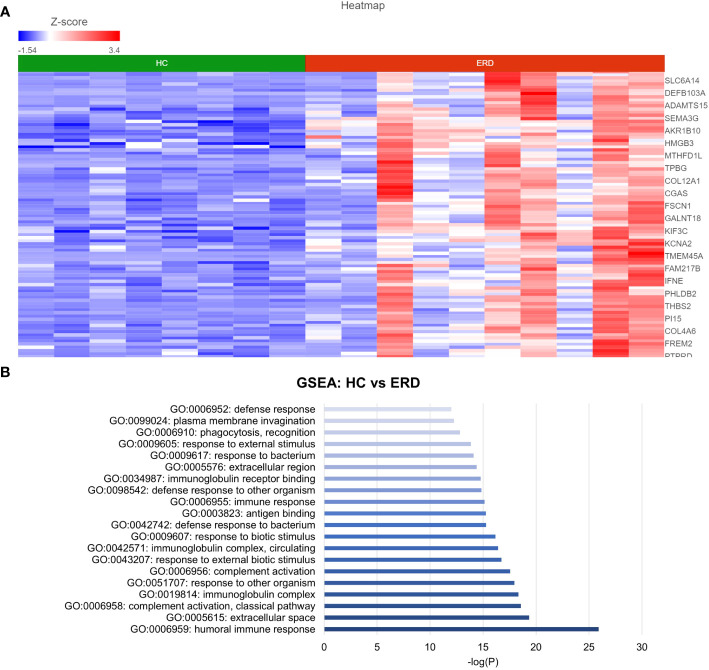
Differentially expressed genes between normal and FH esophageal mucosa. **(A)** Heatmap displaying the FDR-filtered most significantly DE genes between HC and FH from a total of 711 DE genes. *Z* score = log2 fold change in gene expression. **(B)** Bar graph of the most statistically significative functional categories highlighted (*p* < 0.01). HC: *N* = 8, FH: *N* = 8.

**Figure 4:**
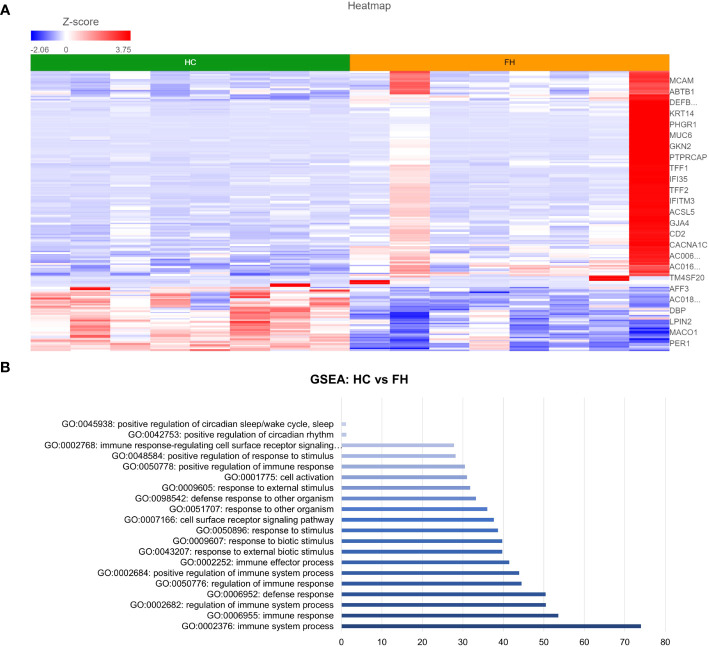
Differential gene expression between ERD and normal esophageal mucosa. **(A)** Heatmap displaying the FDR-filtered top DE genes between HC and ERD from a total of 356 DE genes. *Z* score = log2 fold change in gene expression. **(B)** Bar graph of the most statistically significative functional categories highlighted (*p* < 0.01). HC: *N* = 8, ERD: *N* = 10.

**Figure 5:**
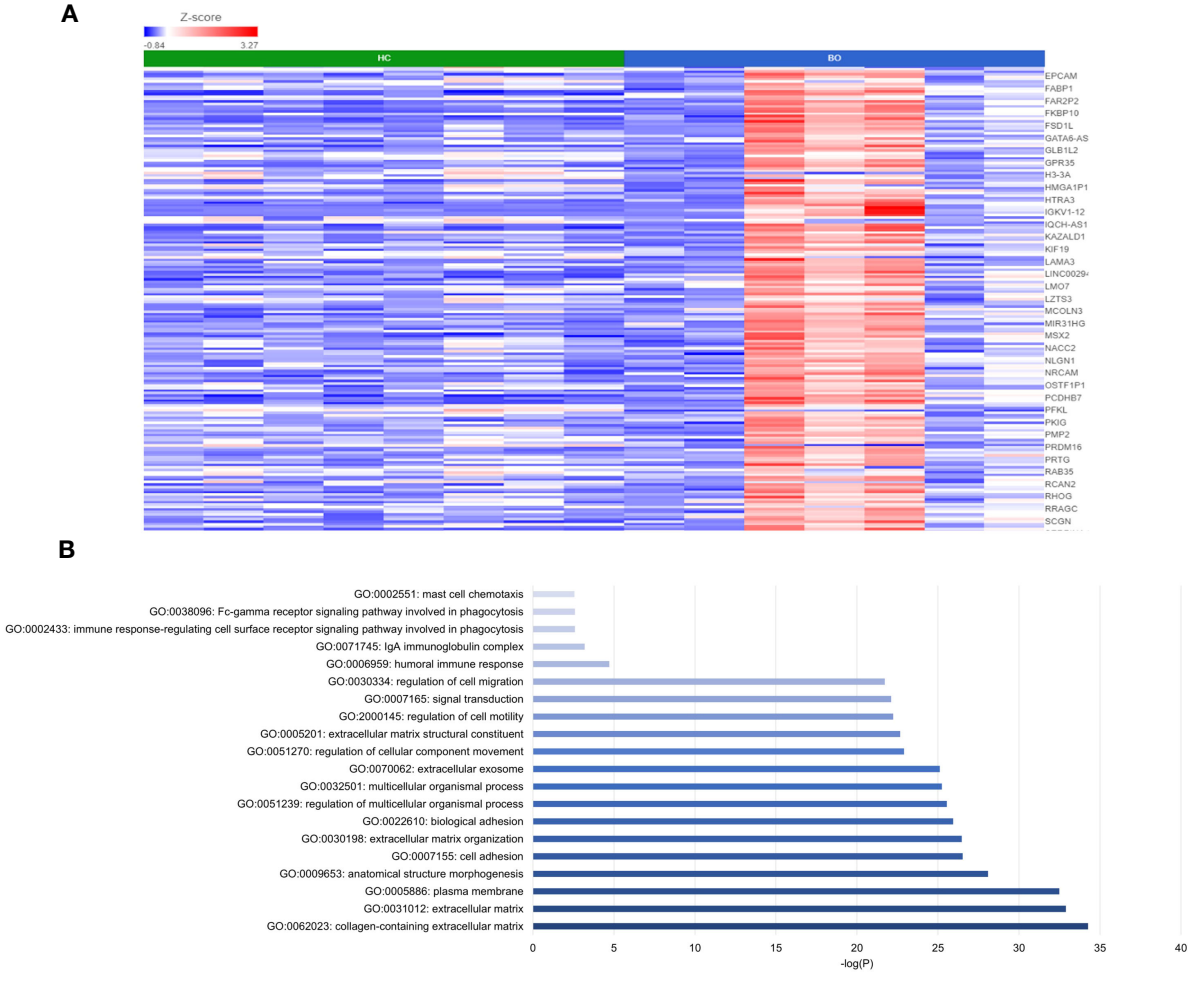
Differential gene expression between normal and BE esophageal mucosa. **(A)** Heatmap displaying the top significantly DE genes between HC and BE from a total of 3,010 DE genes. *Z* score = log2 fold change in gene expression. **(B)** Bar graph of the most statistically significative functional categories highlighted (*p* < 0.01). HC: *N* = 8, BE: *N* = 7.

DESeq2 analysis of HCs with NERD patients alone highlighted the upregulation of genes including *DES*, *GKN1*, *ADAM9*, and *MMP9* in NERD compared with HCs (**Figure 2A**), which were highlighted in the maintenance of gastrointestinal epithelium, epithelial structure maintenance, and regulation of the innate immune response (**Figure 2B**). The mucosal differences between FH patients and HCs were also individually assessed and showed downregulation of circadian rhythm-related genes *PER1* and *CIART*, which were highly expressed in the healthy esophagus (**Figures 3A, B**). Compared with the healthy esophageal mucosa, ERD patients had 356 DE genes including an increased expression of *CXCL1*, *KRT10*, *KRT16*, and *CCL21* and downregulation of the tight junction protein *CLDN10* (**Figure 4A**). Statistically significant DE genes (*p* ≤ 0.01) were highlighted in several molecular pathways including positive regulation of cell proliferation, humoral immune response, and complement activation (**Figure 4B**). DESeq2 analysis of HCs and BE patients highlighted 3,010 DE genes, including *IGKV1-12* and *NRCAM* which were among the most significantly upregulated genes in BE compared with healthy controls, while *MUC21* and *TGM1-2* were among the most significantly downregulated genes in BE (**Figure 5A**). These DE genes were highlighted in biological pathways including igA immunoglobulin complex, extracellular matrix organization, and regulation of cell migration, as shown in **Figure 5B**.

### 3.2 Dendritic cells are more abundant in healthy esophageal mucosa compared with GERD

**Figure 6:**
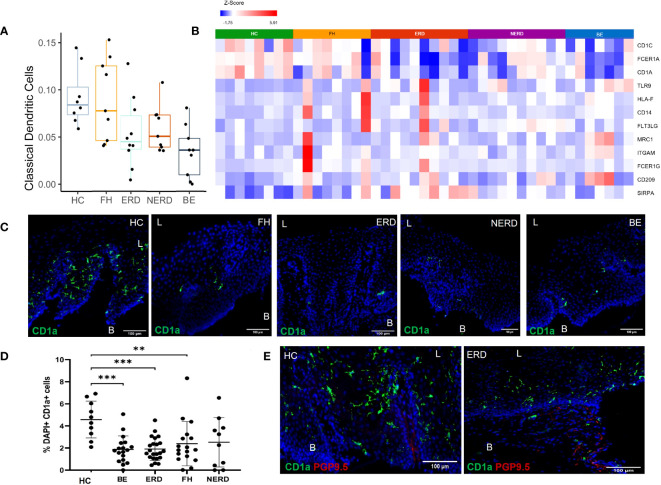
CD1a^+^ dendritic cells are more abundant in the healthy esophageal mucosa than in patients with GERD. **(A)** Boxplot displaying dendritic cell fractions in the esophageal mucosa of patients with GERD and healthy controls. Graph coded manually using R using data output from CIBERSORT. BE: *N* = 9, ERD: *N* = 10, FH: *N* = 8, HC: *N* = 8, NERD: *N* = 9. **(B)** DESeq2 expression matrix for the comparison between HCs and GERD patients was manually filtered for dendritic cell markers. HCs: *N* = 8, BE: *N* = 7, ERD: *N* = 10, FH: *N* = 8, NERD: *N* = 10; *p* < 0.05. **(C)** Interpapillary CD1a^+^ dendritic cells residing in the esophageal mucosa in healthy controls and patients with GERD. Scale bar: 100 μm. **(D)** Quantification of dendritic cells in healthy controls: *N* = 10, BE patients: *N* = 18, ERD: *N* = 22, FH: *N* = 17, NERD: *N* = 10. One-way ANOVA and subsequent Bonferroni’s test revealed significantly higher dendritic cell residence in healthy controls compared with patients with BE (*p* = 0.0005), ERD (*p* = 0.0004), and FH (*p* = 0.0096). Error bars represent SD. **(E)** Interpapillary CD1a^+^ dendritic cells in a representative ERD sample and submucosal PGP9.5^+^ afferent nerves; interpapillary CD1a^+^ dendritic cells and submucosal PGP9.5^+^ afferent nerves in a representative healthy control sample. Scale bar: 100 μm. L, luminal, B, basal aspect of the biopsy sample.

Based on the finding of differential immune-related genes between HCs and GERD esophageal mucosa, we next estimated the proportions of different immune cell types present in our esophageal mucosal tissue sequenced in bulk, using computational deconvolution methods. The relative levels of distinct immune cells within esophageal mucosal RNA isolated from bulk-sequenced FH (*N* = 8), NERD (*N* = 9), ERD (*N* = 10), BE (*N* = 9), and HC (*N* = 8) samples were determined using CIBERSORT and filtered for *p <*0.05 (21). This highlighted decreased dendritic cell fraction in the esophageal mucosa in BE, ERD, and NERD patients compared with FH patients and healthy controls (**Figure 6A**). DESeq2 analysis between healthy controls and GERD patients highlighted significantly higher expression of *CD1C*, *CD1A*, and *FCER1A*, genes encoding monocyte-derived dendritic cell surface markers (22, 23), in healthy controls compared to GERD patients (**Figure 6B**). FH patients showed relatively higher levels of expression of dendritic cell marker genes compared with NERD, ERD, and BE patients (**Figure 6B**).

Immunofluorescence (IF) studies were undertaken to study the expression profile of dendritic cell surface markers highlighted with RNA sequencing to infer posttranslational modifications at the protein level. CD1a, a well-described dendritic cell subset marker, was used to identify dendritic cells present in the esophageal mucosa of biopsies from healthy controls and GERD patients. GERD samples phenotyped into ERD (*N* = 22), NERD (*N* = 10), FH (*N* = 17), and BE (*N* = 18) and healthy controls (*N* = 10) were evaluated for CD1a^+^ dendritic cells in the esophageal mucosa. CD1a^+^ dendritic cells were most frequently interpapillary in nature, being detected on the outside of and in between papillary structures (**Figure 6C**). The abundance of CD1a^+^ dendritic cells was significantly higher in healthy controls compared to BE (*p* = 0.0005), ERD (*p* = 0.0004), and FH patients (*p* = 0.0096) (**Figure 6D**).

The localization of dendritic cells was also assessed in relation to deep afferent nerve endings previously detected in a representative number of ERD samples and healthy controls with PGP9.5. There appeared to be no anatomical relationship between afferent nerves and dendritic cells in the esophageal mucosa. PGP9.5^+^ afferent nerves were detected in the submucosa but were not in close proximity to interpapillary dendritic cells detected in the esophageal mucosa in ERD patients or healthy controls (**Figure 6E**).

### 3.3 Mast cells are closely apposed to afferent nerve endings in ERD

**Figure 7:**
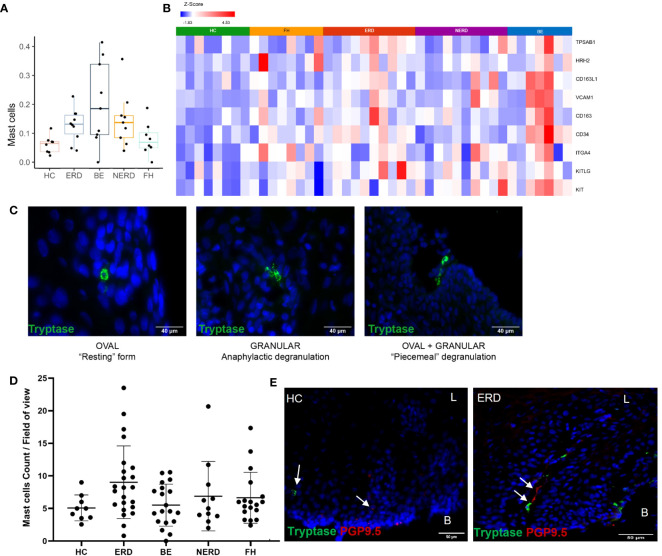
Anatomical localization of mast cells in GERD esophageal mucosa. **(A)** Boxplot displaying mast cell fractions in the esophageal mucosa of patients with GERD and healthy controls. Graph coded manually using R using data output from CIBERSORT. BE: *N* = 9, ERD: *N* = 10, FH: *N* = 8, HC: *N* = 8, NERD: *N* = 9. **(B)** DESeq2 expression matrix for the comparison between HCs and GERD patients was manually filtered for mast cell markers on Partek. HCs: *N* = 8, BE: *N* = 7, ERD: *N* = 10, FH: *N* = 8, NERD: *N* = 10; *p* = 0.05. **(C)** Morphology and activity status of mast cells in the esophageal epithelium of patients with GERD. A mix of mast cell morphologies was often seen in the same representative ERD sample. **(D)** Mast cell infiltration among the disease groups was not statistically different (*p* = 0.0751). **(E)** A representative ERD sample with a deep afferent nerve marked by PGP9.5 in the same mucosal papillae as a mast cell and a representative HC with an arrow of an intrapapillary afferent nerve ending, while a mast cell was detected within the mucosa localized away from the positive PGP9.5 signal. Scale bar: 50 μm. L, luminal, B, basal aspect of the biopsy sample.

Immune enrichment analysis of the RNA sequencing dataset highlighted a higher abundance of mast cells in esophageal mucosal biopsies from patients with NERD, ERD, and BE compared with patients with FH and healthy controls (**Figure 7A**). DESeq2 analysis between healthy controls and GERD patients highlighted significantly higher expression of *TPSAB1*, the gene that encodes tryptase, in ERD patients compared with HCs, and the common mast cell surface markers *KIT*, *CD34*, *VCAM1*, and *CD16* in BE patients compared with HCs (**Figure 7B**) (24, 25). Mast cell chemoattractant genes, including *CXCL6, MUC3A*, and *TGF-β*, were also significantly higher in GERD compared with HCs (**Supplementary Figure 4**).

The localization of mast cells within the esophageal mucosa was assessed using immunofluorescence staining for mast cell tryptase. Mast cells were most often seen surrounding the papillae near the basal layer of the squamous epithelium, but tryptase granules were occasionally also detected around the more superficial layers of the mucosa (**Figure 7C**). Mast cells of three types of morphologies were observed in esophageal mucosa: 1) oval-shaped mast cells with intracellular tryptase granules, in the “resting” form; 2) those with a highly granulated morphology, in anaphylactic degranulation; and 3) a combination of oval-shaped cells which appeared to be releasing tryptase granules or “piecemeal degranulation,” as shown in a representative ERD sample (**Figure 7C**). These cell types were seen across all GERD phenotypes and healthy controls, with no notable morphologic differences or significant changes to mast cell numbers between healthy controls and GERD phenotypes (**Figure 7D**). Moreover, there was no significant correlation between mast cell infiltration and the severity of inflammation in ERD patients (**Supplementary Figure 3**).

To assess whether tissue infiltration of mast cells induces neuroplastic and inflammatory changes in the esophageal mucosa of patients with GERD, IF data were further evaluated to qualitatively assess the spatial relationship between mast cells residing in the esophageal mucosa and afferent nerve endings. This revealed the innervation of deep PGP9.5-immunoreactive afferent nerves in the same esophageal mucosal papillary structures of ERD patients where mast cell infiltration was observed, while in healthy controls where mast cells were detected, afferent nerve endings were not found within the same papillae (**Figure 7E**).

### 3.4 NGF expression on mast cells is increased in GERD

**Figure 8:**
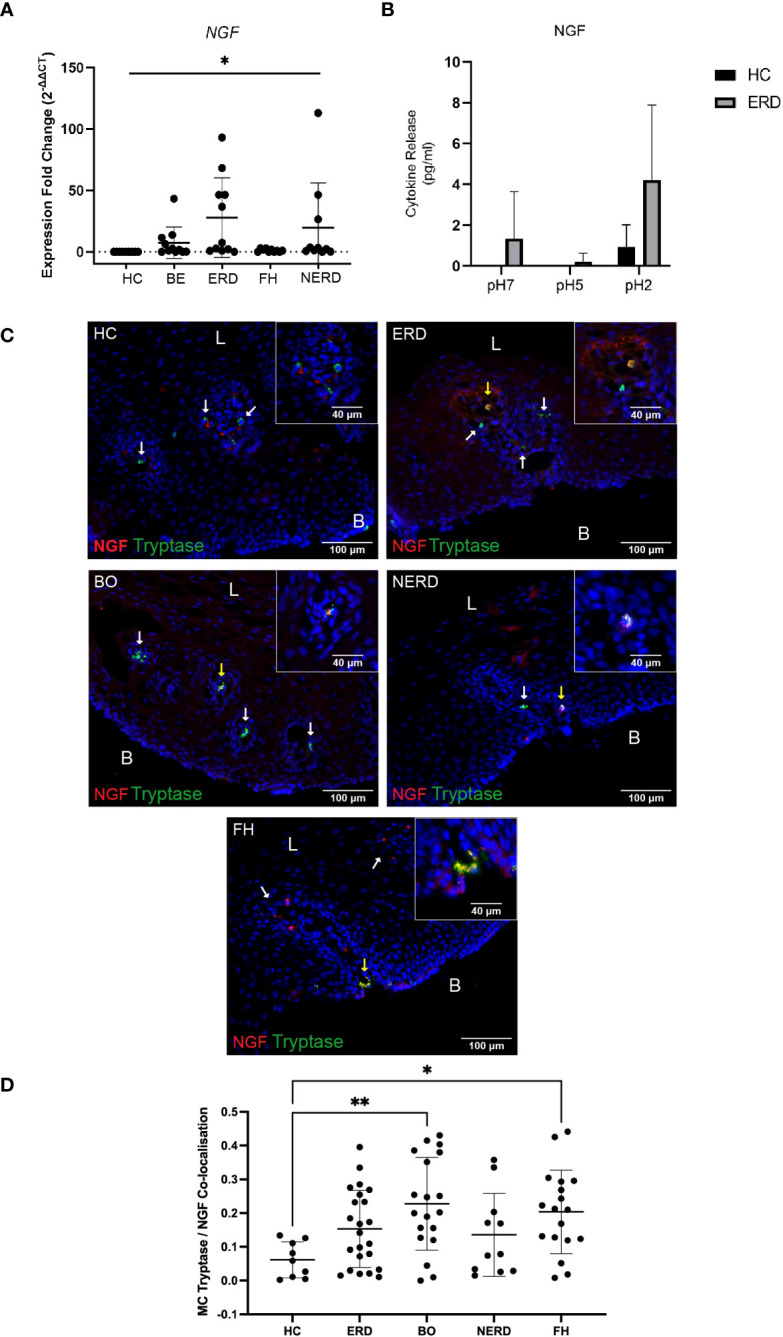
NGF co-expression is increased in mast cells infiltrating the GERD esophageal mucosa. **(A)** Normalized expression fold change of NGF in relation to the reference gene 18S, normalized against healthy control samples. One-way ANOVA detected a significant difference among the control and GERD samples (*p* = 0.03). NERD: *N* = 10, BE: *N* = 11, ERD: *N* = 11, FH: *N* = 10, Control: *N* = 9. **(B)** No significant differences in NGF were observed (*p* > 0.05, two-way ANOVA); three biopsies were taken from the control (*N* = 3) and ERD (*N* = 3). Error bars represent SD. **(C)** Oval-shaped mast cells identified with mast cell tryptase (shown by white arrows) adjacent to the papillae do not express NGF in HC. Released NGF/NGF expressed by other immunocytes near mast cells. Intrapapillary mast cells co-express NGF (yellow arrow) in a patient with ERD. Intrapapillary mast cells co-express NGF (yellow arrow) and mast cell without NGF expression in nearby papillae (white arrow) in a representative BE patient. A deep intrapapillary mast cell was co-expressing NGF (yellow arrow), while another mast cell in close proximity (white arrow) did not express NGF in a patient with NERD. NGF and tryptase colocalization in a degranulating intrapapillary mast cell (yellow arrow) and released NGF detected in the same papillae in a patient with FH. The scale bar represents 100 μm; the insert scale bar represents 40 μm. **(D)** Quantification of colocalization between mast cell tryptase and NGF using Manders’ coefficient (M1) detected significantly higher NGF co-expression in BE (*p* = 0.0094) and FH (*p* = 0.0458) compared with HCs. Error bars represent SD. L, luminal side, B, basal aspect of the biopsy sample.

To assess whether mast cell infiltration into the esophageal mucosa of patients with GERD causes neuroplastic changes through NGF release, the relative level of *NGF* gene expression in the esophageal mucosa of patients with GERD was assessed via qPCR studies. *NGF* gene expression was higher in GERD esophageal mucosa compared with healthy esophageal mucosa (*p* = 0.03) (**Figure 8A**). ERD and NERD patients had the highest level of *NGF* gene expression among the GERD phenotypes although the difference between ERD/NERD samples and healthy controls did not reach statistical significance (*p* = 0.09 and *p* = 0.66, respectively) (**Figure 8A**). *Ex-vivo* biopsy experiments assessing esophageal mucosal response to acid exposure also detected a noticeably increased NGF release from ERD esophageal mucosal biopsies at baseline, pH 5, and pH 2 compared with healthy control esophageal mucosa (**Figure 8B**).

GERD samples phenotyped into ERD (*N* = 23), BE (*N* = 19), NERD (*N* = 11), and FH (*N* = 18) and healthy controls (*N* = 9) were assessed for NGF protein expression on mast cells infiltrating the esophageal mucosa. Tryptase^+^ mast cells detected in the esophageal mucosa were found to frequently co-express NGF in all GERD groups, and NGF^+^Tryptase^+^ mast cells were intrapapillary (**Figure 8C**).

Quantitative analysis demonstrated that GERD patients had significantly higher NGF co-expressing on mast cells compared with healthy controls (**Figure 8D**) (*p* = 0.0087). *Post-hoc* analysis with Bonferroni’s test detected significantly higher NGF/tryptase colocalization in patients with BE (*p* = 0.0094) and patients with FH (*p* = 0.0458) compared with healthy controls (**Figure 8D**).

## 4 Discussion

**Figure 9:**
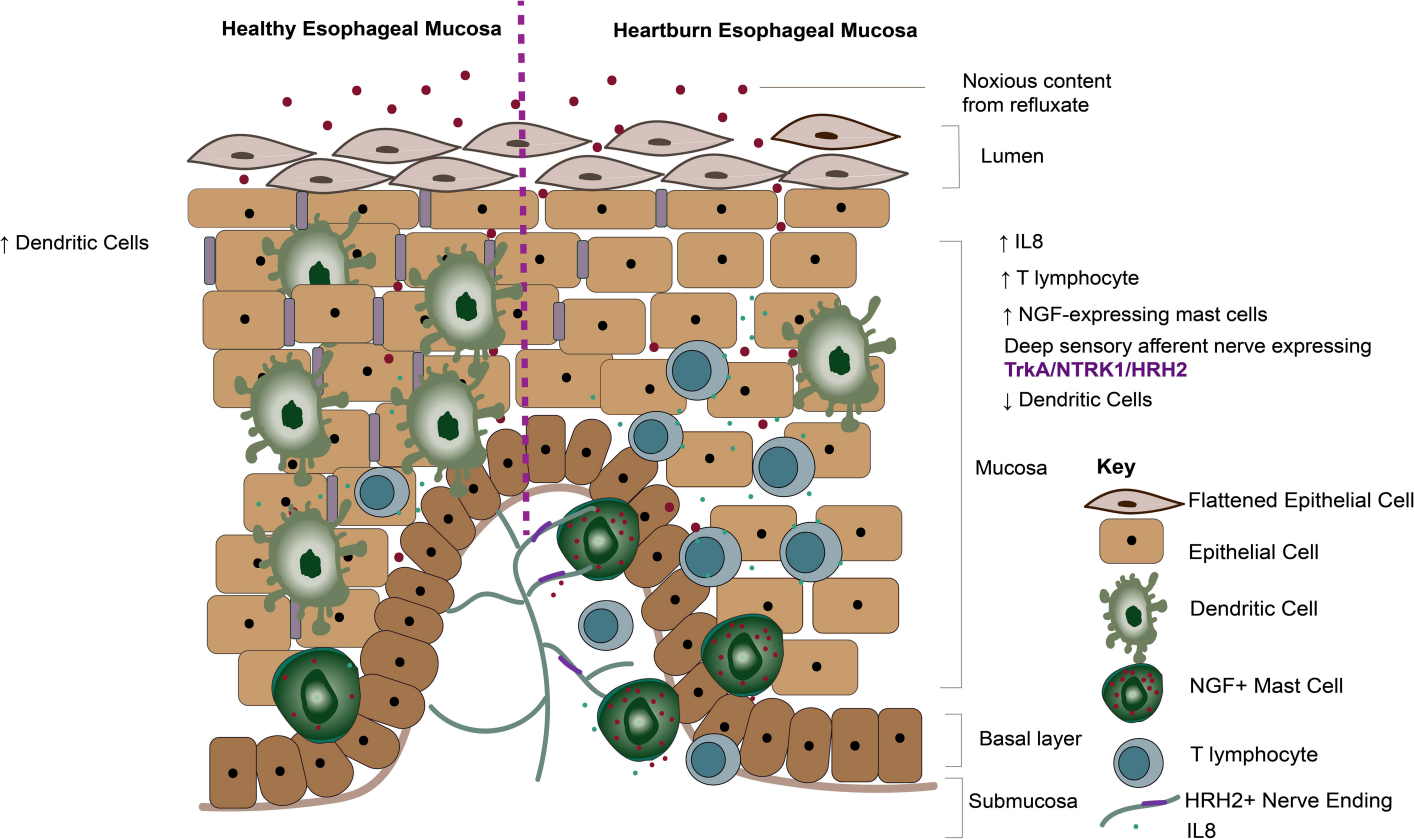
Summary diagram of the findings. Diagram demonstrating mucosal mechanisms elucidated by our current study. Dendritic cells are significantly more abundant in healthy esophageal mucosa compared with GERD phenotypes. In contrast, dendritic cells are decreased, while NGF-expressing mast cells are increased in heartburn patients. Histamine receptor 2 and NTRK1 expressed on deep sensory nerves in close apposition to mast cells likely get sensitized by neuropeptides such as NGF released by mast cells.

In this study, we report phenotypic transcriptomic changes to the immune gene signature across the chronic GERD disease spectrum, concurring with the role of T-lymphocyte infiltration driving acute inflammation at ERD onset (26). We demonstrate a significant loss in dendritic cell infiltration in the GERD esophageal mucosa compared with healthy esophageal mucosa. We also report a close association between deep afferent nerve endings and mast cells in ERD patients, with increased NGF expression on mast cells infiltrating the GERD esophageal mucosa. Our data suggest a connection between neuropeptides and mucosal inflammation in reflux sensation, as summarized by a schematic model in **Figure 9**.

A decreased mucosal dendritic cell population in GERD patients suggests a pathogenic change in the GERD esophageal mucosa. It is likely that immature dendritic cells in the healthy esophageal mucosa, a squamous epithelium exposed to antigens from the lumen, survey the epithelium for pathogens to release anti-inflammatory cytokines to suppress an adaptive immune response. Dendritic cells observed in the esophageal mucosa were often interpapillary, suggesting that they reside in the epithelial layer rather than infiltrate from the submucosa, as often seen with other immune cells (26, 27). Our findings suggest that the oral tolerance mechanism was significantly compromised in patients with FH, ERD, and BE and considerably decreased in patients with NERD. However, the insignificant difference between healthy controls and NERD patients was possibly due to the lower sample number in this group of patients compared with the other GERD phenotypes. These findings are similar to those reported in the literature in the intestine, where intestinal dendritic cells are integral for preventing pathological immune responses to harmless antigens (28–31). Our findings of dendritic cell loss in GERD, coupled with increased infiltration of mast cells, likely highlight a switch to the adaptive immune response in GERD, where other immune cell populations are recruited to protect the esophageal mucosa against luminal antigens (26). There did not seem to be an association between esophageal dendritic cells screening the mucosa and afferent nerve endings seen in healthy controls or GERD patients, suggesting that the mechanism of dendritic cell immune response in GERD is not sensory nerve-driven.

The increased mast cell population compared with healthy controls, as suggested by cellular deconvolution of our transcriptomic data, may result in increased hypersensitivity experienced by ERD patients. Although IF studies failed to detect a significant difference in mast cell infiltration between GERD samples and healthy controls, the median number of mast cells detected by mast cell tryptase staining was notably higher in ERD and BE compared with HCs. Mast cells have been widely recognized for their role in initiating reciprocal communication with nociceptors on sensory nerve fibers in a range of inflammatory conditions (32–35). Being one of the first responders of the immune system present near externally exposed surfaces allows sensitized nerves to signal to tissue-resident innate immune cells like mast cells by releasing inflammatory mediators such as substance P which can activate mast cells to release neurotrophic factors including NGF, leading to a bidirectional cycle of hypersensitivity (36). The observation of intrapapillary mast cells in very close apposition to deep afferent nerve endings in the papillae of patients with ERD further supports this as a potential mechanism that may lead to increased pain transmission in a group of patients who do not present with superficial afferent nerve innervation. This might be due to the histamine released from mast cells which may induce activation of sensory fibers by interacting with histamine receptors on nerve endings, based on our finding of *HRH2* gene encoding histamine receptor 2 upregulation in GERD compared with HCs.

We detected a notable increase in the median mast cell quantification in the hyposensitive BE cohort compared with HCs. However, sensitivity is a complex mechanism generated by multiple factors including neuronal innervation, receptor expression, and their interaction with inflammatory mediators. It is also important to note that the BE samples included in our study were from the squamous tissue above the BE segment; thus, the sensory profile of Barrett’s itself remains relatively unstudied.

There is increased co-expression of NGF in mast cells infiltrating the esophageal mucosa of patients with GERD compared with healthy controls, which may be one of the key mechanisms behind heartburn sensation. Elevated mast cell numbers and NGF content characterize a number of inflammatory conditions including the colonic mucosa in IBD patients (37–39). The increase in NGF content in mast cells infiltrating the esophageal mucosa of GERD patients could be a possible mechanism of nerve fiber sprouting leading to sensitivity. Overexpression of NGF in the dorsal horn of the adult spinal cord has previously been associated with extensive axonal sprouting, where the axons were identified as a subpopulation of nociceptive fibers expressing CGRP and substance P, suggesting that NGF induces neuronal plasticity and regulates the hyperalgesic response (40). NGF regulates nerve fiber outgrowth and, thus, pain transmission by signaling through its tyrosine kinase receptor A (NTRK1). A recent study importantly highlighted increased nerve fiber density and sprouting and increased expression of NGF on tryptase^+^ mast cells in mucosal colon tissues from IBS patients compared with controls, highlighting a role for NGF in increasing nerve sprouting by signaling via NTRK1 receptors expressed on nerve fibers (32). Our findings suggest that mucosal mast cells are also key players in heartburn transmission, given their increased expression of NGF which likely leads to sprouting of nociceptive nerve endings, thus increasing activation of sensory pathways. The morphology of intrapapillary mast cells detected near mucosal afferent nerves in ERD patients being “anaphylactic” further suggests that they could be releasing NGF, histamine, or proteases which lower the activation threshold of the nerve endings and perpetuate esophageal sensitivity.

This study has several strengths including demonstrating novel transcriptomic findings on esophageal mucosal expression of neuroimmune markers which were validated on corresponding patient biopsies immunohistochemically. Additionally, the patients included in the study are carefully phenotyped by endoscopic and reflux studies. A limitation of the study is that patients with BE included in the study were “on” PPIs at the time of endoscopy, unlike ERD, NERD, and FH patients who discontinued PPI use at least 7 days prior to biopsy collection. This was in keeping with clinical guidelines which issue long-term PPI administration for BE patients to reduce the risk of esophageal adenocarcinoma (41). Moreover, the healthy control group was considerably younger than the GERD group, which may have played a role in the mucosal findings of the study. Reflux perception is multifactorial, and other comorbidities, such as stress and the use of medications other than antireflux that were not excluded, could conceivably impact the mucosa. Finally, we acknowledge that biopsies represent only a small percentage of esophageal surface area, thus limiting the number of nerve endings detected and potentially influencing spatial relationships between mast cells and nerve endings.

We believe that this study has advanced the current understanding of mucosal pathogenesis of GERD and has translational potential. In ERD patients where superficial sensory nerves were not identified, NGF-expressing mast cells in close vicinity to deep afferent nerves are likely to have an indirect role in inducing pain transmission upon NGF release into the esophageal mucosa and subsequent activation of neighboring nerves. The loss of dendritic cells in heartburn patients could conceivably highlight a switch to the adaptive immune response, where other immune cell populations are recruited to protect the esophageal mucosa against luminal antigens. Our findings raise the enticing possibility of topical therapy with antagonists against NGF and histamine receptors, particularly in patients who are refractory to PPIs, and warrant further mechanistic experiments to further unravel potential therapeutic targets.

In conclusion, our study has identified two key findings in well-phenotyped GERD patients. First, there is a loss in conventional dendritic cell populations in heartburn patients compared with healthy subjects. This is likely the first step in the inflammatory response seen in the GERD esophageal mucosa preceding the activation of the adaptive immune response. Second, mast cells infiltrating the GERD esophageal epithelium have increased NGF co-expression. This may highlight a peripheral sensitization mechanism driving a sensory change in the perception of acid reflux stimuli. These findings suggest that immune cell regulation may reduce symptom generation in the treatment of GERD patients, perhaps in the form of novel topical antagonists. These findings warrant follow-up studies for further elucidation of peripheral sensitization mechanisms to discover more robust treatment targets.

## Data availability statement

The sequencing data discussed in this publication are deposited in the NCBI’s Gene Expression Omnibus and are accessible through GEO Series accession number GSE226303.

## Ethics statement

The studies involving humans were approved by the NRES Committee London-Queensquare (Study reference: 19/LO/1506). The studies were conducted in accordance with the local legislation and institutional requirements. The participants provided their written informed consent to participate in this study.

## Author contributions

AU: Data curation, Formal Analysis, Investigation, Methodology, Validation, Writing – original draft, Writing – review & editing. FD: Data curation, Formal Analysis, Visualization, Writing – review & editing. MD’a: Data curation, Investigation, Visualization, Writing – review & editing. SM: Formal Analysis, Visualization, Writing – review & editing. CL: Funding acquisition, Methodology, Project administration, Resources, Writing – review & editing. EM: Project administration, Resources, Writing – review & editing. DB: Methodology, Supervision, Visualization, Writing – review & editing. DK: Conceptualization, Methodology, Supervision, Writing – review & editing. DS: Funding acquisition, Project administration, Resources, Supervision, Writing – review & editing. PW: Conceptualization, Funding acquisition, Methodology, Supervision, Visualization, Writing – review & editing. MP: Conceptualization, Investigation, Methodology, Supervision, Visualization, Writing – review & editing.
