# Supplementary material for: Identification of novel immune cell signature in gastroesophageal reflux disease: altered mucosal mast cells and dendritic cell profile
Source: Front Immunol. 2023 Nov 30;14:1282577. doi: 10.3389/fimmu.2023.1282577 (PMC10720318; doi:10.3389/fimmu.2023.1282577)
Supplement: Supplementary file 1 [file DataSheet_1.docx]

**Table A.1: Clinical and demographic data for patient cohort**

Table includes RDQ score, LA grade for patients with ERD, and total acid exposure time (AET) where clinical data was available for recruited patients.

| Sample ID | Phenotype | Gender | Age | Ethnicity | RDQ Score | LA grade | Total AET (%) | Studies |
| --- | --- | --- | --- | --- | --- | --- | --- | --- |
| AE161118 | NERD | Male | 19 | White British | 40 | n/a | n/a | IF, qPCR, RNA-seq |
| JF110219 | ERD | Male | 45 | White British | 30 | 2 | n/a | IF, qPCR |
| RQ110219 | NERD | Female | 70 | White British | 16 | n/a | 15.5 | IF, qPCR |
| JG120219 | ERD | Male | 60 | White British | n/a | 3 | n/a | IF, qPCR |
| AG140219 | NERD | Male | 40 | Indian | 12 | n/a | 8 | IF, qPCR |
| MS161118 | ERD | Male | 58 | White other | 12 | 4 | n/a | IF, qPCR |
| AG200219 | FH | Male | 42 | White British | 28 | n/a | 3.4 | IF, qPCR, RNA-seq |
| DS200219 | NERD | Male | 54 | Other | 60 | 1 | 7 | IF, qPCR, RNA-seq |
| JM010319 | ERD | Male | 32 | Other | 5 | 3 | n/a | IF, qPCR, RNA-seq |
| JH050319 | FH | Male | 69 | White British | 45 | n/a | n/a | IF, qPCR |
| BA060319 | NERD | Male | 54 | White British | 15 | n/a | 14.1 | IF, qPCR, RNA-seq |
| JU130319 | FH | Female | 37 | White British | 54 | n/a | Physiological | IF, qPCR, RNA-seq |
| SSG130319 | ERD | Male | 39 | Other | 57 | 2 | n/a | IF, qPCR, RNA-seq |
| JD220319 | ERD | Male | 50 | Other | 0 | 2 | n/a | IF, qPCR |
| EM230519 | BO | Male | 61 | White other | 12 | n/a | n/a | IF, qPCR |
| CB120419 | FH | Female | 72 | White British | 58 | n/a | 1.3 | IF, qPCR, RNA-seq |
| EB120419 | FH | Female | 33 | Bangladeshi | 40 | n/a | 3 | IF, qPCR, RNA-seq |
| RM120419 | FH | Male | 34 | Bangladeshi | 38 | n/a | 4 | IF, qPCR, RNA-seq |
| SC160519 | NERD | Female | 46 | White other | 47 | n/a | 6.6 | IF, qPCR, RNA-seq |
| BT150519 | ERD | Male | 48 | White British | 33 | 2 | n/a | IF, qPCR |
| LJ300519 | NERD | Female | 52 | Indian | 24 | n/a | n/a | IF, qPCR, RNA-seq |
| RM250619 | ERD | Male | 46 | White British | 8 | 3 | n/a | IF, qPCR, RNA-seq |
| AO190719 | NERD | Female | 52 | Black | 0 | n/a | n/a | IF, qPCR, RNA-seq |
| JW140819 | BO | Male | 29 | White British | 29 | 1 | n/a | IF, qPCR, RNA-seq |
| MK210819 | NERD | Female | 32 | White British | 32 | n/a | 13.4 | IF, qPCR |
| SV210819 | NERD | Female | 39 | Indian | 16 | n/a | 6.3 | IF, qPCR, RNA-seq |
| ML290819 | FH | Female | 58 | n/a | 60 | n/a | 3.7 | IF, qPCR |
| DS120919 | NERD | Male | 29 | n/a | 8 | 3 | n/a | IF, qPCR, RNA-seq |
| WB081019 | ERD | Male | 40 | White British | 27 | 2 | n/a | IF, qPCR, RNA-seq |
| PB091019 | BO | Female | 61 | White British | 0 | n/a | n/a | IF, qPCR, RNA-seq |
| LK091019 | BO | Female | 73 | White British | 0 | n/a | n/a | IF, qPCR, RNA-seq |
| SM211019 | BO | Female | 36 | Other | 38 | n/a | n/a | IF, qPCR |
| BA221019 | BO | Female | 67 | White British | 0 | n/a | n/a | IF, qPCR, RNA-seq |
| CF231019 | BO | Female | 55 | Other | 28 | 1 | n/a | IF, qPCR, RNA-seq |
| SH061119 | BO | Female | 57 | n/a | 0 | n/a | n/a | IF, qPCR, RNA-seq |
| SG131119 | FH | Male | 53 | Ethiopian | n/a | n/a | n/a | IF, qPCR, RNA-seq |
| JI050220 | NERD | Female | 54 | Arab | 27 | n/a | 8.4 | IF, qPCR, RNA-seq |
| SM050220 | ERD | Female | 73 | White British | 11 | 4 | n/a | IF, qPCR, RNA-seq |
| GK190220 | BO | Male | 37 | Greek | 33 | n/a | n/a | IF, qPCR, RNA-seq |
| CB050320 | ERD | Male | 19 | White British | 27 | 3 | n/a | IF, qPCR, RNA-seq |
| JB110320 | BO | Male | 54 | White Irish | 6 | n/a | n/a | IF, qPCR, RNA-seq |
| RC110320 | BO | Male | 73 | White British | 0 | n/a | n/a | IF, qPCR, RNA-seq |
| SF030320 | ERD | Male | 56 | White British | 0 | 2 | n/a | IF |
| CB050320 | ERD | Male | 19 | White British | 27 | 3 | n/a | Functional assay |
| SJ010921 | ERD | Female | 33 | White British | 38 | 3 | n/a | Functional assay |
| SF270921 | ERD | Female | 76 | White British | 25 | 3 | n/a | Functional assay |
| HF040320 | HC | Female | 34 | White British | 0 | 0 | n/a | IF, qPCR, RNA-seq |
| MV120521 | HC | Female | 35 | White British | 0 | 0 | n/a | IF, qPCR, RNA-seq |
| BB260521 | HC | Male | 25 | White other | 0 | 0 | n/a | IF, qPCR, RNA-seq |
| HD090621 | HC | Male | 70 | White British | 0 | 0 | n/a | IF, qPCR |
| AV140721 | HC | Female | 25 | Indian | 0 | 0 | n/a | IF, qPCR, RNA-seq |
| ZP280721 | HC | Female | 20 | White European | 0 | 0 | n/a | IF, qPCR, RNA-seq |
| MG050821 | HC | Female | 24 | White European | 0 | 0 | n/a | IF, qPCR, RNA-seq |
| CL180821 | HC | Female | 26 | Asian | 0 | 0 | n/a | IF, qPCR, RNA-seq |
| TG010921 | HC | Male | 26 | White British | 0 | 0 | n/a | IF, qPCR, RNA-seq |
| SZ290921 | HC | Male | 20 | South Asian | 0 | 0 | n/a | IF, qPCR |
| NU061021 | HC | Female | 25 | White other | 0 | 0 | n/a | Functional Assay |
| MRM201021 | HC | Female | 27 | White British | 0 | 0 | n/a | Functional Assay |
| RD271021 | HC | Male | 27 | Black | 0 | 0 | n/a | Functional Assay |
| MA031121 | HC | Male | 45 | South Asian | 0 | 0 | n/a | IF, qPCR |


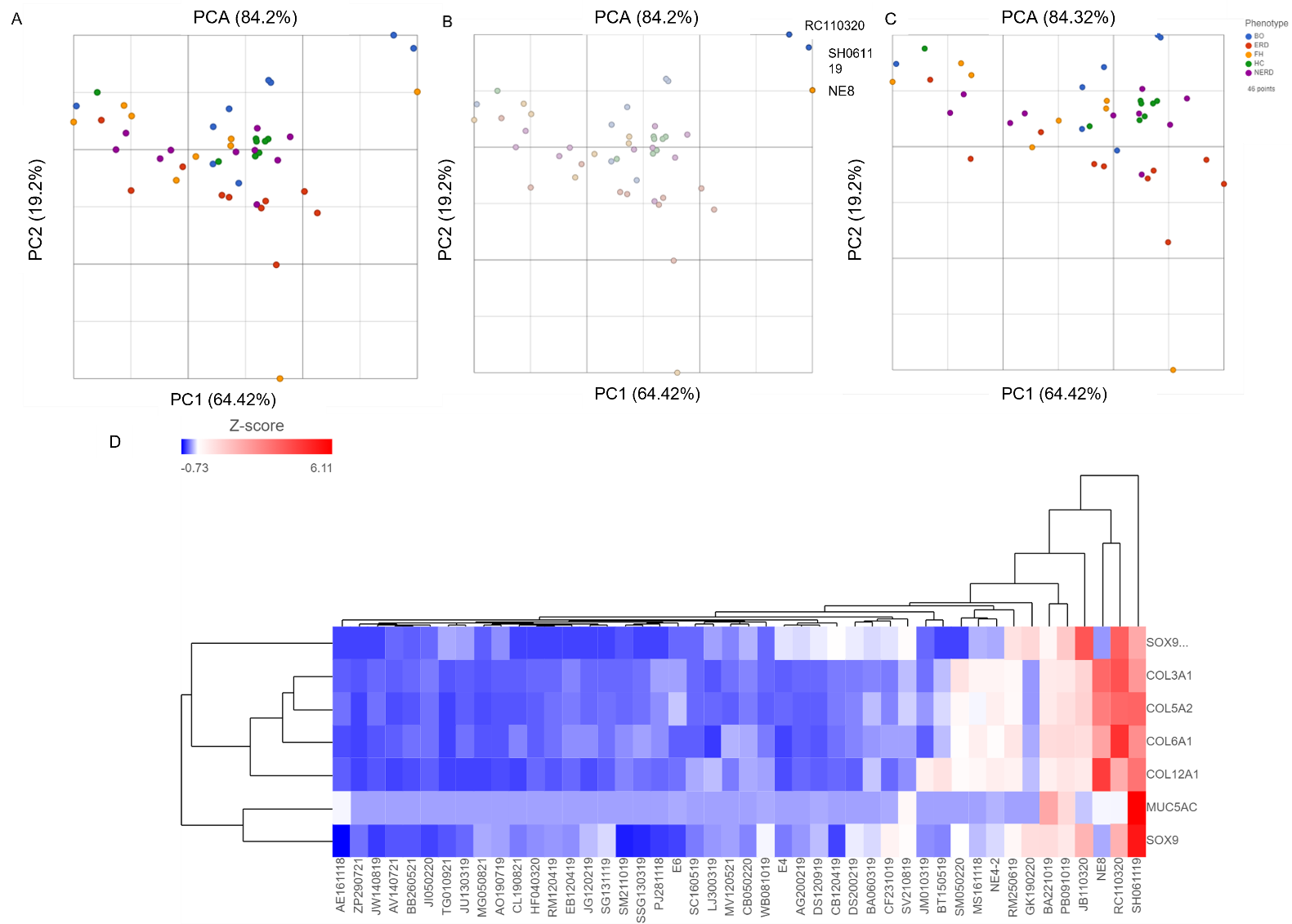


**Fig. 1 PCA plot of expression similarity between samples**

A) PCA plot showing a scatter plot of samples along the first two principal components, where the spatial arrangement of the points reflects data similarity between samples. B) Three outliers highlighted from the PCA: NE8 (FH), SH061119 (BO), and RC110320 (BO). C) Refined PCA with outliers removed based on biological characteristics, i.e. high expression of stromal collagen and Barrett’s segment genes. D) Samples filtered for expression of stromal collagen genes and Barrett’s segment genes. NE8 (FH) had significantly higher expression of stromal collagens COL5A, while samples SH061119 (BO), and RC110320 (BO) had significantly higher expression of stromal collagen genes and Barrett’s segment genes SOX9 and MUC5AC.


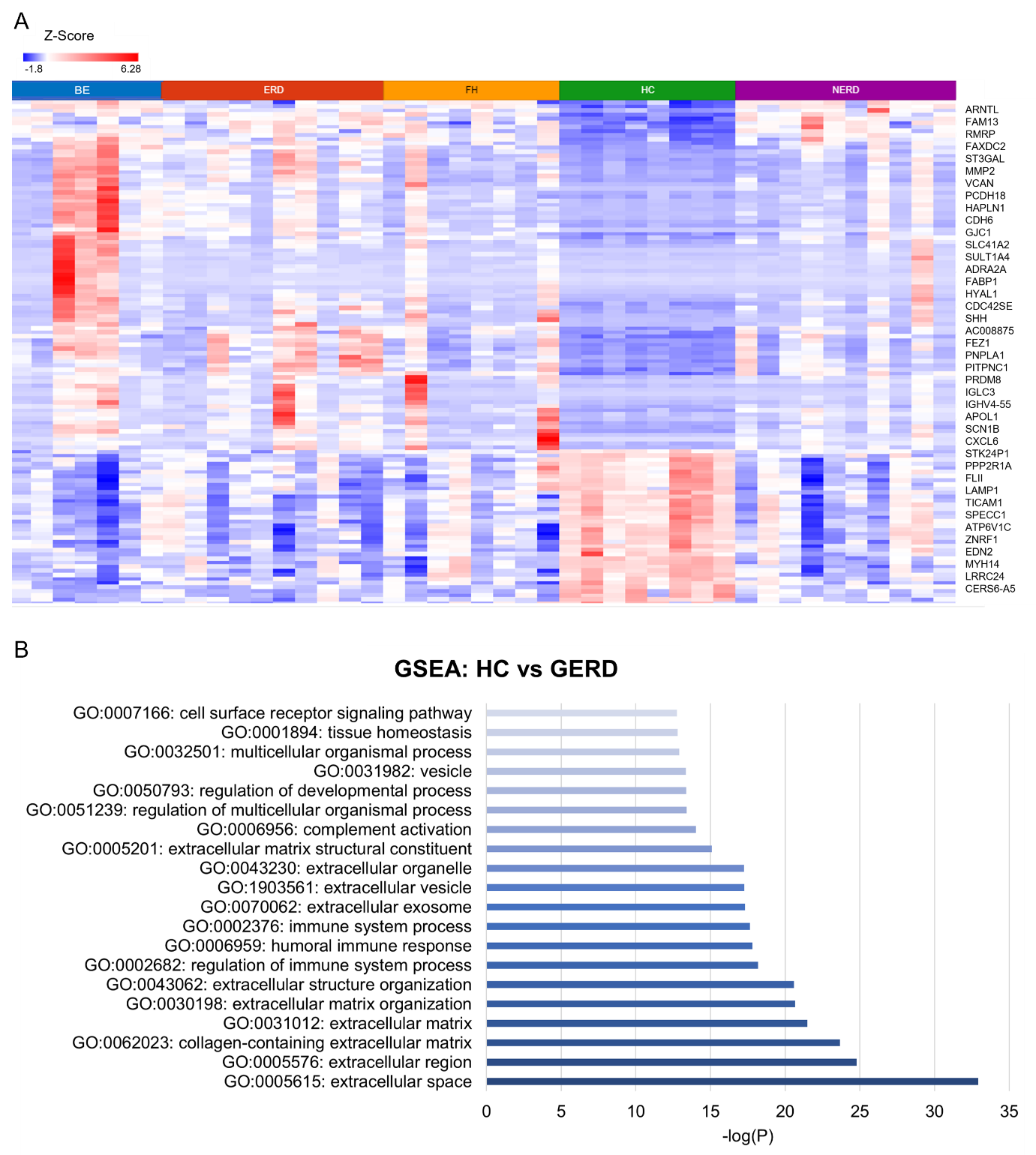


**Fig. 2 Differentially Expressed Genes Related to Structural Barrier Integrity**

A) Heatmap displaying the most significantly DE genes between healthy controls and GERD with FDR *p*=0.01. B) Bar graph displaying the most biologically enriched gene ontology (GO) pathways from GSEA as log scaled *p* values (*p*<0.01). HC: N=8, BO: N=7, ERD: N=10, FH: N=8, NERD: N=9

**Figure 6 Differential Gene Expression Between Normal and BO Oesophageal Mucosa**

Heatmap displaying the top significantly DE genes between HC and BO from a total of 251 genes upregulated and 1978 genes downregulated in HCs compared to BO patients HC: N=8, BO: N=7.


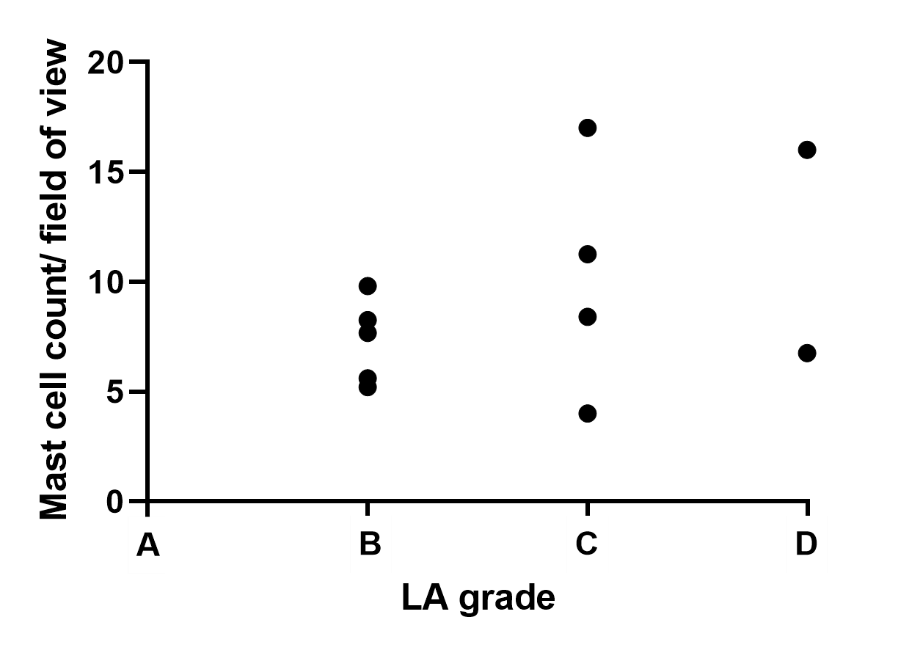


**Fig. 3 Correlation of Mast Cell Quantification and Degree of Inflammation in ERD**

No correlation was found between LA grade of ERD and mast cells quantified per field of view (Logistic regression: *p* = 0.34). ERD: N=11.


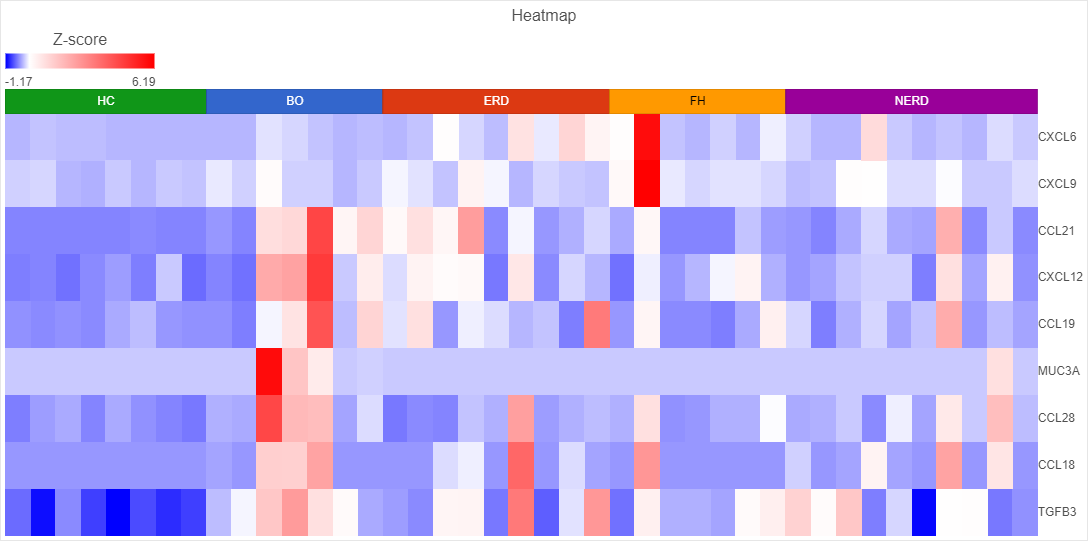


**Fig. 4: Mast Cell Chemoattractants Detected by Transcriptomic Data**

Heatmap highlighting mast cell chemoattractants significantly differentially expressed in GERD compared to healthy controls.
